# Traumatic Stress, Chronic Ethanol Exposure, or the Combination, Alter Cannabinoid System Components in Reward and Limbic Regions of the Mouse Brain

**DOI:** 10.3390/molecules26072086

**Published:** 2021-04-06

**Authors:** Veronica M. Piggott, Scott C. Lloyd, James I. Matchynski, Shane A. Perrine, Alana C. Conti

**Affiliations:** 1 Research & Development Service, John D. Dingell VA Medical Center, Detroit, MI 48201, USA; veronica.piggott@wayne.edu (V.M.P.); sclloyd@med.wayne.edu (S.C.L.); jamatchy@med.wayne.edu (J.I.M.); sperrine@med.wayne.edu (S.A.P.); 2 Department of Psychiatry and Behavioral Neurosciences, Wayne State University School of Medicine, Detroit, MI 48201, USA; 3 Translational Neuroscience Program, Wayne State University School of Medicine, Detroit, MI 48201, USA

**Keywords:** post-traumatic stress disorder, mouse single-prolonged stress, chronic intermittent ethanol, anandamide, 2-arachidonoylglycerol, fatty acid amide hydrolase, monoacylglycerol lipase, reward pathway, limbic system

## Abstract

The cannabinoid system is independently affected by stress and chronic ethanol exposure. However, the extent to which co-occurrence of traumatic stress and chronic ethanol exposure modulates the cannabinoid system remains unclear. We examined levels of cannabinoid system components, anandamide, 2-arachidonoylglycerol, fatty acid amide hydrolase, and monoacylglycerol lipase after mouse single-prolonged stress (mSPS) or non-mSPS (Control) exposure, with chronic intermittent ethanol (CIE) vapor or without CIE vapor (Air) across several brain regions using ultra-high-performance liquid chromatography tandem mass spectrometry or immunoblotting. Compared to mSPS-Air mice, anandamide and 2-arachidonoylglycerol levels in the anterior striatum were increased in mSPS-CIE mice. In the dorsal hippocampus, anandamide content was increased in Control-CIE mice compared to Control-Air, mSPS-Air, or mSPS-CIE mice. Finally, amygdalar anandamide content was increased in Control-CIE mice compared to Control-Air, or mSPS-CIE mice, but the anandamide content was decreased in mSPS-CIE compared to mSPS-Air mice. Based on these data we conclude that the effects of combined traumatic stress and chronic ethanol exposure on the cannabinoid system in reward pathway regions are driven by CIE exposure and that traumatic stress affects the cannabinoid components in limbic regions, warranting future investigation of neurotherapeutic treatment to attenuate these effects.

## 1. Introduction

Individuals who experience traumatic event(s), such as terrorism, combat, witnessing death or a life-threatening injury, the sudden death of loved ones, physical or sexual assault (both adult and child), serious accidents, life-threatening illness, and natural disasters, are at risk of developing post-traumatic stress disorder (PTSD) [1,2,3,4]. PTSD has a lifetime prevalence of approximately 6.8% among adults in the United States [5]. Individuals who have PTSD often use alcohol to ease their PTSD symptoms, and the common co-occurrence of alcohol use disorder (AUD) complicates the treatment of PTSD [6,7,8].

Traumatic stress and alcohol exposure impair the function of the primary structures of the limbic system, including the hippocampus (HC) and amygdala (AMYG), which play important roles in memory and emotion [9,10,11,12,13,14,15,16], as well as the primary structures in the reward pathway, including the prefrontal cortex (PFC), striatum (STR), and nucleus accumbens (NAc), which mediate motivation, reward, and executive function [11,12,17,18,19,20,21]. Although these brain regions differ in their primary function, they are similar in their enriched content of cannabinoid (CB) system components [17,22,23,24], suggesting a role for the CB system in the combined condition of PTSD-AUD.

The CB system is a neuromodulatory network composed of endogenous endocannabinoids and cannabinoid 1 and 2 receptors (CB1Rs and CB2Rs). CB signaling is involved in the regulation of the stress response, emotional states, learning and memory, fear extinction, and reward behavior [25,26,27,28,29,30,31]. The endogenous CB receptor ligands, anandamide (AEA) and 2-arachidonoylglycerol (2-AG), are synthesized and released from postsynaptic neurons to act retrogradely on presynaptic CB receptors [25,32]. In general, AEA and 2-AG regulate synaptic plasticity by acting on presynaptic CB1Rs [25]. AEA is synthesized by N-acyl phosphatidylethanolamine phospholipase (NAPE-PLD) and hydrolyzed by fatty acid amide hydrolase (FAAH), whereas 2-AG is synthesized by α or β isoforms of diacylglycerol lipase (DAGL) and hydrolyzed by multiple enzymes, primarily by monoacylglycerol lipase (~85%) (MAG-L), but also FAAH (~1%), α,β-hydrolase 6 (~4%), α,β-hydrolase 12 (~9%), and others (~1%) [33,34]. A growing body of literature has shown that stress and alcohol exposure alone affect the central CB system [20,23,24,30,35,36,37,38]. Dysfunction of the CB system is associated with the pathophysiology of drug use and mood disorders [39].

Clinical studies have investigated blood levels of AEA and 2-AG in those diagnosed with and without PTSD. Hill et al. showed that the AEA content was increased, while the 2-AG content was decreased, in PTSD patients’ plasma compared to individuals who were exposed to trauma but did not develop PTSD [40]. However, another study showed that both AEA and 2-AG content in plasma increased in patients with PTSD compared to healthy controls [41]. These clinical studies indicate that traumatic stress dysregulates the endocannabinoids, but not consistently. In pre-clinical studies, acute restraint stress consistently resulted in decreased AEA content with increased FAAH activity in the AMYG [24,42,43] as well as in the HC [24,44,45,46], but showed no effect in the medial PFC [24,43,46,47,48] in rodents. On the other hand, acute restraint stress increased 2-AG content in the medial PFC [24,48] and HC [24,45] but had no effect on the AMYG [24,42,47,49]. Chronic stress, such as restraint and social defeat, in rodents caused AEA content to decrease with an associated increase in FAAH activity, and 2-AG content to increase with decreased MAG-L expression in the AMYG [24,47,49,50,51]. Chronic restraint stress in mice also increased AEA content and decreased 2-AG content in the NAc [24,47]. In both medial PFC and HC, chronic restraint and social defeat stress in rodents decreased AEA content and increased 2-AG content [24,44,49,51]. These pre-clinical studies indicate that restraint stress causes decreased amygdalar AEA levels and increased 2-AG levels, with the effects being region-, not duration-dependent.

Ethanol exposure has also been shown to disrupt the CB system. For example, mice exposed to chronic ethanol vapor for 72 h without withdrawal exhibited increased cortical AEA content as well as down-regulated CB1R expression and function in the cortex, STR, HC, and basolateral AMYG, compared to control mice [52]. With a similar paradigm, chronic ethanol vapor exposure for two days increased 2-AG content in the dorsolateral STR [53]. Another study showed that basolateral amygdalar AEA content decreased, while 2-AG content was not changed after rats were exposed to chronic ethanol vapor for 6 weeks. This study also demonstrated that AEA content was not changed in the ventral medial PFC, while 2-AG content was decreased [54]. An alcohol liquid diet study showed that rats exposed to alcohol for 24 h showed a decrease in striatal AEA, but not 2-AG compared to rats given a water diet [55]. In a clinical study, basal plasma AEA content was lower, while the 2-AG content did not change, in alcoholics compared to healthy social drinkers [56]. These studies provide evidence that ethanol exposure affects cortical, striatal, and amygdalar CB system in a duration-dependent manner.

Our previous study showed that traumatic stress combined with acute alcohol administration decreased CB1R levels in the mouse anterior STR compared to control groups [20]. Our previous reports have also shown that traumatic stress combined with chronic intermittent ethanol vapor exposure and withdrawal uniquely increased ethanol consumption compared to the control group (non-stress, air exposure) in mice [57]. However, no animal studies have been reported to examine the effects of traumatic stress combined with chronic ethanol exposure on the brain levels of the endocannabinoid ligands (AEA and 2-AG), their synthetic enzymes (NAPE-PLD and α-DAGL), their degradatory-hydrolytic enzymes (FAAH and MAG-L), and CB1R. Therefore, the aim of this study was to combine the use of traumatic stress and chronic ethanol vapor exposure mouse models to examine AEA and 2-AG contents as well as NAPE-PLD, α-DAGL, FAAH, MAG-L, and CB1R levels in the PFC, anterior STR, NAc, dorsal HC, and AMYG. Based on previous literature, we hypothesized that in the main reward-related brain regions (PFC, STR, NAc), AEA level would increase and cause FAAH and CB1R levels to decrease with unchanged 2-AG, NAPE-PLD, α-DAGL, and MAG-L levels in mice exposed to both traumatic stress and chronic ethanol vapor compared to controls. However, in the primary structures of the limbic brain regions (HC and AMYG), we hypothesized that AEA levels would decrease and cause FAAH to increase, and 2-AG levels would decrease and cause MAG-L to increase with unchanged NAPE-PLD, α-DAGL, and CB1R in mice exposed to both traumatic stress and chronic ethanol vapor compared to controls.

## 2. Results

**Figure 1:**
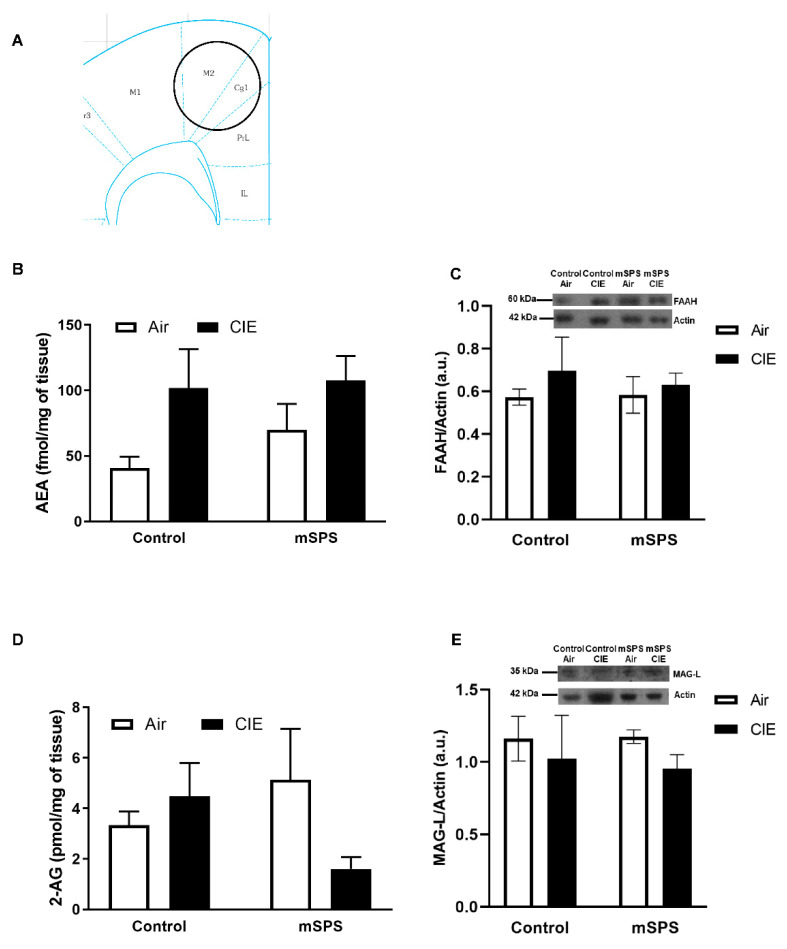
Anandamide (AEA) and 2-arachidonoylglycerol (2-AG) contents, fatty acid amide hydrolase (FAAH) and monoacylglycerol lipase (MAG-L) levels in the prefrontal cortex (PFC) after mSPS/Control or Air/CIE exposures. (**A**) Schematic of coronal slice where PFC tissue punches were taken bilaterally (adapted from [58]). Average (**B**) AEA content (Control-Air: n = 4; Control CIE: n = 7; mSPS-Air: n = 6; mSPS-CIE: n = 8) (**C**) FAAH levels (Control-Air: n = 6; Control CIE: *n* = 4; mSPS-Air: n = 5; mSPS-CIE: n = 8) (Inset: representative immunoblotting sample images; kDa kilodaltons) (**D**) 2-AG content (Control-Air: n = 4; Control CIE: n = 7; mSPS-Air: n = 6; mSPS-CIE: n = 8), and average (**E**) MAG-L levels (Control-Air: n = 7; Control CIE: n = 4; mSPS-Air: n = 4; mSPS-CIE: n = 7) in the PFC (Inset: representative immunoblotting sample images) did not change among groups after mSPS/Control or Air/CIE exposures. Data are mean ± SEM.

UPLC-MS/MS was used to measure AEA and 2-AG content, and immunoblotting was used to measure NAPE-PLD, DAGL, FAAH, MAG-L, and CB1R levels in the PFC, anterior STR, NAc, dorsal HC, and AMYG. Protein levels of NAPE-PLD, DAGL, and CB1R did not change among groups across the brain regions examined in this study (see Figures S1–S3 in Supplementary Materials). Samples were excluded if their statistic values were greater than the critical value using the two-sided Grubbs’ test for outlier analysis. Figure 1 illustrates the portion of the PFC being studied (Figure 1A) and shows average AEA (Figure 1B), FAAH (Figure 1C), 2-AG (Figure 1D), and MAG-L (Figure 1E) levels following treatment. Two samples were unrecoverable during the AEA and 2-AG extraction procedure. Two samples from the MAG-L level analysis, and one sample from the FAAH level analysis were excluded after outlier analysis. A two-way ANOVA showed no interaction of stress and/or ethanol on AEA or 2-AG, MAG-L, or FAAH levels in the PFC after Control/mSPS or Air/CIE exposures. However, this analysis revealed an ethanol main effect (F_(1,21)_ = 8.0; *p* = 0.010) on AEA levels. A post hoc Tukey HSD analysis (Control-Air: n = 4; Control CIE: n = 7; mSPS-Air: n = 6; mSPS-CIE: n = 8) showed that AEA content did not change between groups.

**Figure 2:**
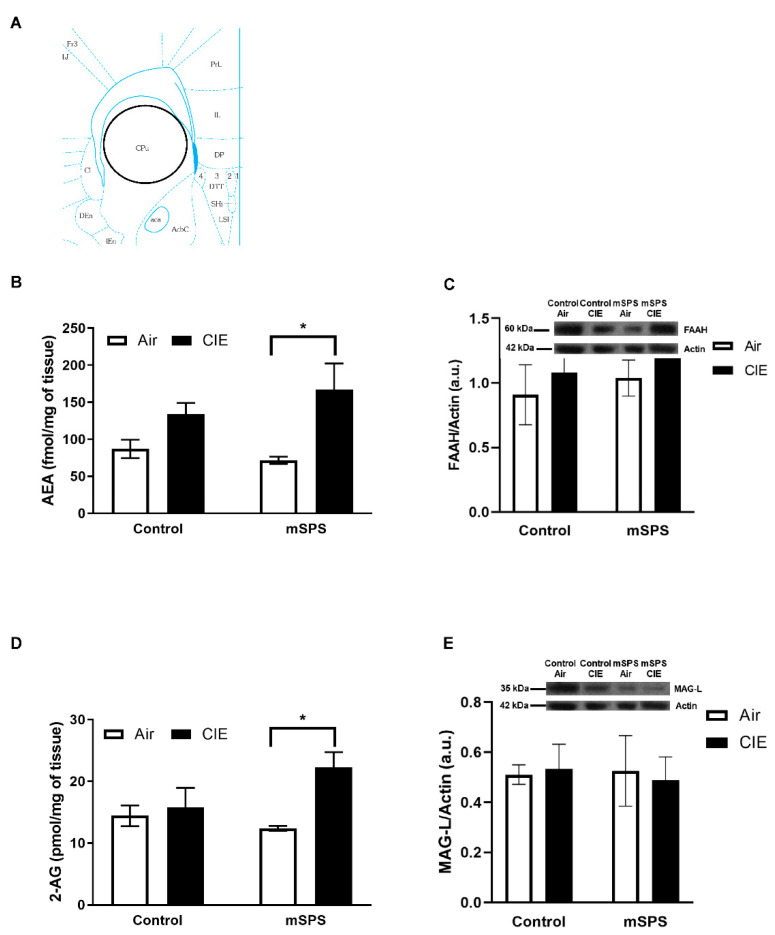
Anandamide (AEA) and 2-arachidonoylglycerol (2-AG) contents, fatty acid amide hydrolase (FAAH) and monoacylglycerol lipase (MAG-L) levels in the anterior striatum (STR) after mSPS/Control or Air/CIE exposures. (**A**) Schematic of coronal slice where anterior STR tissue punches were taken bilaterally (adapted from [58]). Average (**B**) AEA content (Control-Air: n = 6; Control CIE: n = 7; mSPS-Air: n = 6; mSPS-CIE: n = 8) in the anterior STR was significantly increased in mSPS-CIE mice compared to mSPS-Air mice (* *p* < 0.05). However, average (**C**) FAAH level (Control-Air: n = 7; Control CIE: n = 4; mSPS-Air: n = 5; mSPS-CIE: n = 8) did not change among groups (Inset: representative immunoblotting sample images). Average (**D**) 2-AG content (Control-Air: n = 6; Control CIE: n = 6; mSPS-Air: n = 5; mSPS-CIE: n = 8) in the anterior STR was significantly increased in mSPS-CIE mice compared to mSPS-Air mice (* *p* < 0.05), but the average (**E**) MAG-L levels (Control-Air: n = 7; Control CIE: n = 4; mSPS-Air: n = 5; mSPS-CIE: n = 8) did not change among groups (Inset: representative immunoblotting sample images). Data are mean ± SEM.

Figure 2 illustrates the portion of the anterior STR being studied (Figure 2A) and shows average AEA (Figure 2B), FAAH (Figure 2C), 2-AG (Figure 2D), and MAG-L (Figure 2E) levels following treatment. Two samples from the 2-AG content analysis were excluded after outlier analysis. A two-way ANOVA revealed no interaction of mSPS and/or CIE on AEA or 2-AG, or FAAH or MAG-L levels. However, this analysis showed an ethanol main effect on the AEA (F_(1,23)_ = 9.32; *p* = 0.006) and 2-AG (F_(1,21)_ = 5.46; *p* = 0.029) levels. A post hoc Tukey HSD analysis revealed AEA levels in the anterior STR was significantly increased in mSPS-CIE mice (n = 8) compared to mSPS-Air mice (n = 6; *p* = 0.034), and 2-AG levels was also significantly increased in mSPS-CIE mice (n = 8) compared to mSPS-Air mice (n = 5; *p* = 0.037).

**Figure 3:**
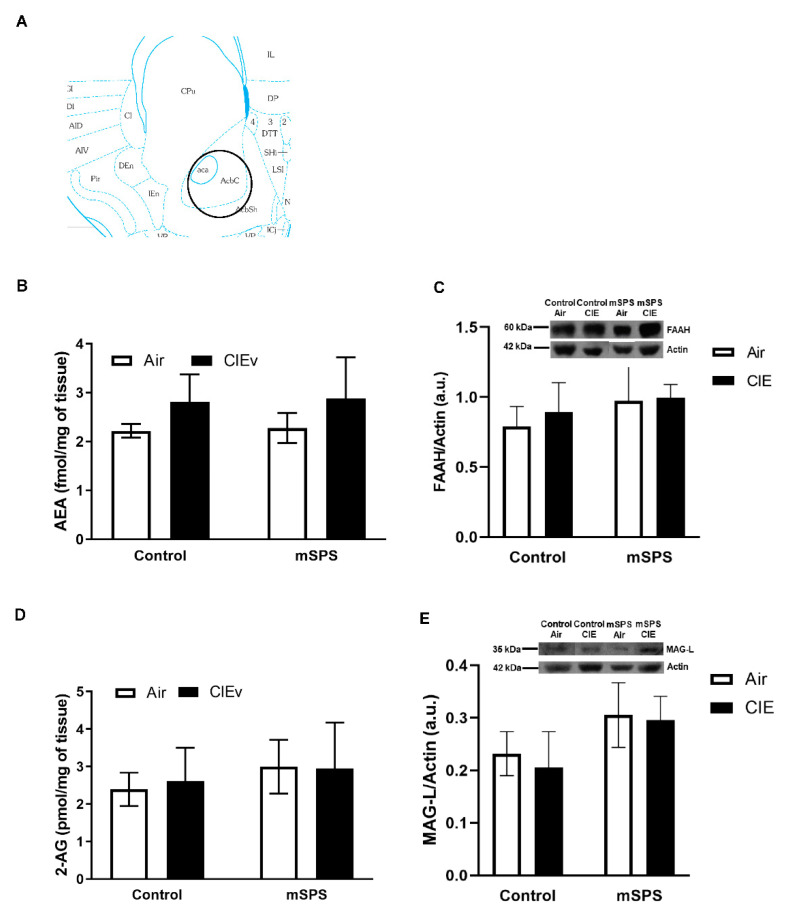
Anandamide (AEA) and 2-arachidonoylglycerol (2-AG) contents, fatty acid amide hydrolase (FAAH) and monoacylglycerol lipase (MAG-L) levels in the nucleus accumbens (NAc) after mSPS/Control or Air/CIE exposures. (**A**) Schematic of coronal slice where NAc tissue punches were taken bilaterally (adapted from [58]). Average (**B**) AEA content (Control-Air: n = 3; Control CIE: n = 5; mSPS-Air: n = 6; mSPS-CIE: n = 6) and (**C**) FAAH levels (Control-Air: n = 7; Control CIE: n = 4; mSPS-Air: n = 5; mSPS-CIE: n = 8) (Inset: representative immunoblotting sample images) did not change among groups after mSPS/Control or Air/CIE exposures. The average (**D**) 2-AG content (Control-Air: n = 3; Control CIE: n = 6; mSPS-Air: n = 6; mSPS-CIE: n = 7) and the average (**E**) MAG-L levels (Control-Air: n = 7; Control CIE: n = 4; mSPS-Air: n = 5; mSPS-CIE: n = 8) (Inset: representative immunoblotting sample images) did not change among groups after mSPS/Control or Air/CIE exposures. Data are mean ± SEM.

Figure 3 shows the portion of the NAc being studied (Figure 3A) and revealed average AEA (Figure 3B), FAAH (Figure 3C), 2-AG (Figure 3D), and MAG-L (Figure 3E) levels following treatment. Four samples were unrecoverable during the AEA and 2-AG extraction procedure. Two samples from the AEA content analysis were excluded after outlier analysis. A two-way ANOVA showed no interaction of mSPS and/or CIE on AEA, 2-AG, FAAH, or MAG-L levels in the NAc after Control/mSPS or Air/CIE exposures. This analysis also showed no mSPS or CIE main effect on the AEA or 2-AG content, or FAAH or MAG-L levels in the NAc.

**Figure 4:**
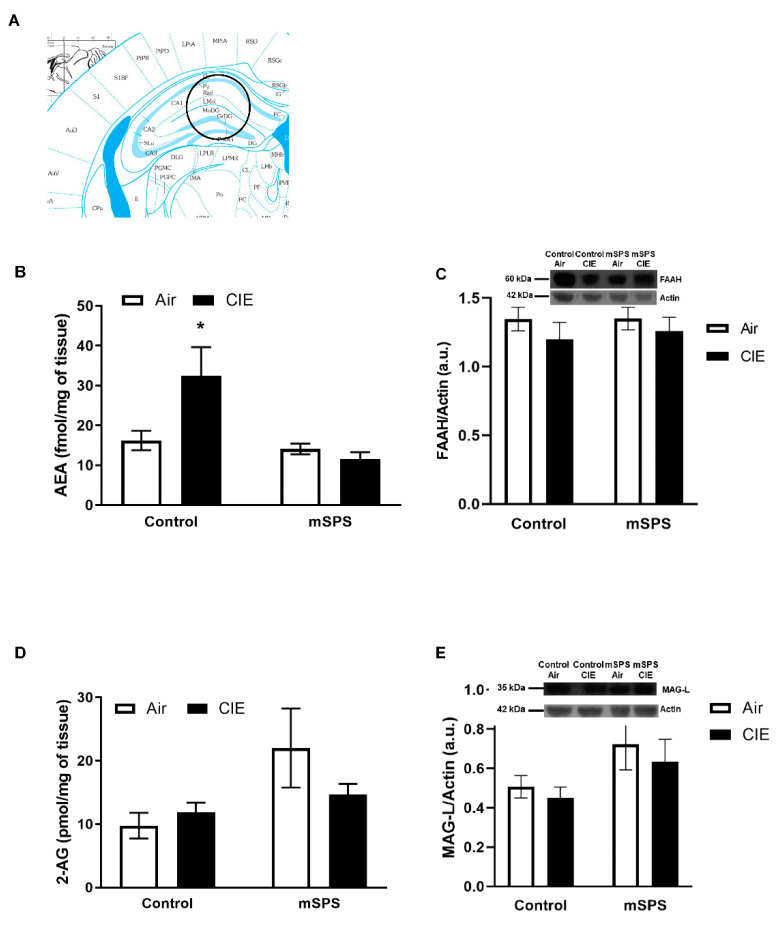
Anandamide (AEA) and 2-arachidonoylglycerol (2-AG) contents, fatty acid amide hydrolase (FAAH) and monoacylglycerol lipase (MAG-L) levels in the dorsal hippocampus (HC) after mSPS/Control or Air/CIE exposures. (**A**) Schematic of coronal slice where dorsal HC tissue punches were taken bilaterally (adapted from [58]). Average (**B**) AEA content (Control-Air: n = 6; Control CIE: n = 7; mSPS-Air: n = 6; mSPS-CIE: n = 8) increased in Control-CIE mice compared to Control-Air, mSPS-Air, or mSPS-CIE mice after mSPS/Control or Air/CIE exposures (* *p* < 0.05). However, the average (**C**) FAAH levels (Control-Air: n = 6; Control CIE: n = 4; mSPS-Air: n = 5; mSPS-CIE: n = 8) (Inset: representative immunoblotting sample images) did not change among groups after mSPS/Control or Air/CIE exposures. In addition, average (**D**) 2-AG (Control-Air: n = 6; Control CIE: n = 7; mSPS-Air: n = 6; mSPS-CIE: n = 8) and (**E**) MAG-L levels (Control-Air: n = 6; Control CIE: n = 4; mSPS-Air: n = 5; mSPS-CIE: n = 8) (Inset: representative immunoblotting sample images) did not change among groups after mSPS/Control or Air/CIE exposures. Data are mean ± SEM.

Figure 4 shows the portion of the dorsal HC being studied (Figure 4A), and illustrates average AEA (Figure 4B), FAAH (Figure 4C), 2-AG (Figure 4D), and MAG-L (Figure 4E) levels following treatment. One sample from FAAH and MAG-L levels analysis was excluded after outlier analysis. A two-way ANOVA showed an mSPS and ethanol interaction (F_(1,23)_ = 5.26; *p* = 0.031) on the AEA content but not on 2-AG, FAAH, or MAG-L levels. This analysis also showed an mSPS main effect on AEA (F_(1,23)_ = 7.91; *p* = 0.010) or 2-AG (F_(1,23)_ = 5.62; *p* = 0.027) levels. A post hoc Tukey HSD analysis revealed that the AEA content in the dorsal HC was increased in Control-CIE mice (n = 7) compared to Control-Air (n = 6; *p* = 0.049), mSPS-Air (n = 6, *p* = 0.022) or mSPS-CIE (n = 8; *p* = 0.005) mice. However, the post hoc Tukey HSD analysis showed no significant difference among groups on 2-AG content.

**Figure 5:**
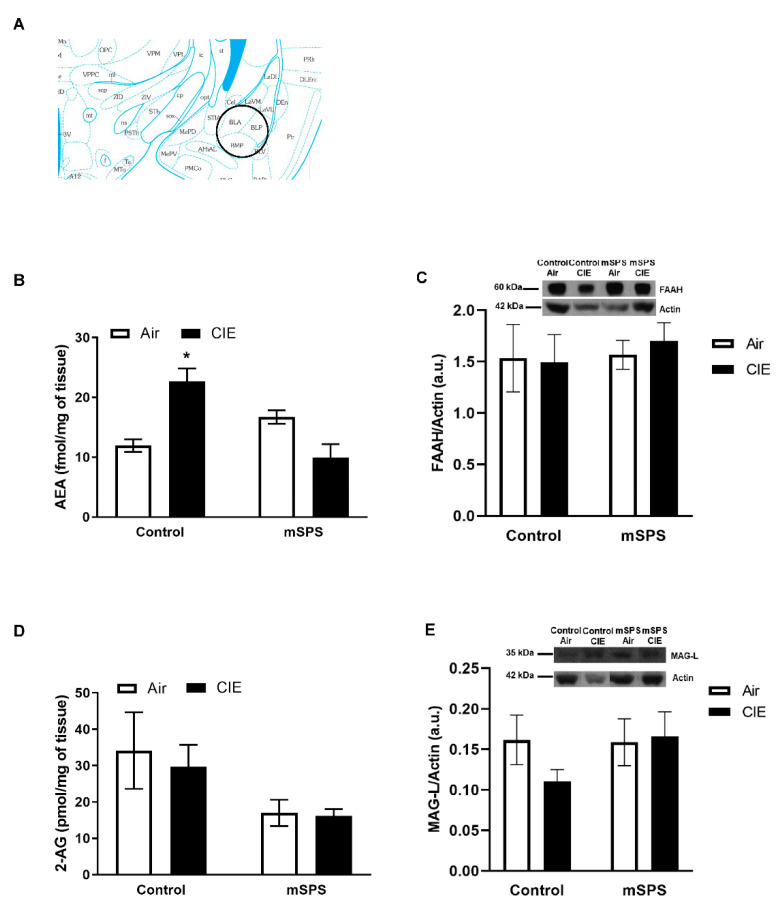
Anandamide (AEA) and 2-arachidonoylglycerol (2-AG) contents, fatty acid amide hydrolase (FAAH) and monoacylglycerol lipase (MAG-L) levels in the amygdala (AMYG) after mSPS/Control or Air/CIE exposures. (**A**) Schematic of coronal slice where AMYG tissue punches were taken bilaterally (adapted from [58]). Average (**B**) AEA content (Control-Air: n = 6; Control CIE: n = 6; mSPS-Air: n = 6; mSPS-CIE: n = 8) increased in Control-CIE mice (* *p* < 0.05) compared to Control-Air or mSPS-CIE mice after mSPS/Control or Air/CIE exposures. However, the average (**C**) FAAH levels (Control-Air: n = 7; Control CIE: n = 4; mSPS-Air: n = 5; mSPS-CIE: n = 8) (Inset: representative immunoblotting sample images) did not change among groups after mSPS/Control or Air/CIE exposures. In addition, average (**D**) 2-AG content (Control-Air: n = 6; Control CIE: n = 6; mSPS-Air: n = 6; mSPS-CIE: n = 7) and (**E**) MAG-L levels (Control-Air: n = 6; Control CIE: n = 3; mSPS-Air: n = 5; mSPS-CIE: n = 8) (Inset: representative immunoblotting sample images) did not change among groups. Data are mean ± SEM.

Figure 5 illustrates the portion of the AMYG being studied (Figure 5A) and shows average AEA (Figure 5B), FAAH (Figure 5C) and 2-AG (Figure 5D), and MAG-L (Figure 5E) levels following treatment. One sample was unrecoverable during the AEA and 2-AG extraction procedure. One sample from the 2-AG content analysis, and two samples from the MAG-L level analysis were excluded after outlier analysis. A two-way ANOVA showed an mSPS and ethanol interaction (F_(1,22)_ = 22.43; *p* = 0.0001) on the AEA content but not on 2-AG, FAAH, or MAG-L levels. This analysis also showed an mSPS main effect on AEA (F_(1,22)_ = 4.65; *p* = 0.042) or 2-AG (F_(1,21)_ = 6.18; *p* = 0.021) levels. A post hoc Tukey HSD analysis revealed that the AEA content in the AMYG was increased in Control-CIE mice (n = 6) compared to Control-Air (n = 6; *p* = 0.003) or mSPS-CIE (n = 8; *p* = 0.0004) mice. For the 2-AG content, a post hoc Tukey HSD analysis showed no difference in 2-AG among groups.

## 3. Discussion

The current study evaluated levels of AEA and 2-AG and their degradatory-hydrolytic enzymes in the PFC, anterior STR, NAc, dorsal HC, and AMYG after mice were exposed to either traumatic stress, chronic ethanol vapor, or a combination of those conditions. The hypothesis of this study was that mice exposed to both mSPS and CIE would have higher AEA content as a result of lower FAAH and CB1R levels, and unchanged 2-AG, NAPE-PLD, α-DAGL, and MAG-L levels in the main reward-associated brain regions studied here (PFC, anterior STR, and NAc) compared to controls. We also hypothesized that mice exposed to both mSPS and CIE would have lower AEA content caused by higher FAAH levels and increased 2-AG content resulting in lower MAG-L levels with unchanged NAPE-PLD, α-DAGL, and CB1R levels in the primary structures of the limbic-associated brain regions studied here (dorsal HC and AMYG) compared to controls. We found that AEA and 2-AG contents in mouse PFC did not change among groups. However, we found that AEA and 2-AG contents were increased in the anterior STR of mSPS-CIE mice compared to mSPS-Air mice, and AEA and 2-AG content in mouse NAc did not change among groups. In dorsal HC, AEA content increased in Control-CIE mice compared to Control-Air, mSPS-Air, or mSPS-CIE mice, and 2-AG content did not change among groups. In AMYG, AEA content increased in Control-CIE mice compared to Control-Air or mSPS-CIE mice. However, amygdalar 2-AG content did not change among groups. Finally, neither mSPS, CIE nor these co-occurring conditions affected NAPE-PLD, α-DAGL, MAG-L, FAAH or CB1R levels compared to controls in any brain region examined.

In this study, we focused on the main brain regions of the reward pathway (PFC, anterior STR, NAc) as well as the two primary brain structures of the limbic system (HC and AMYG) as traumatic stress and chronic alcohol exposure are known to disrupt the function of those circuits [17,18,19,59,60,61], and brain regions within those pathways contain abundant levels of CB system components [17,22,23,24]. The PFC is one of the main reward-associated brain regions, and it plays an important role in working memory, executive function, emotional and motivational regulation, learning extinction, and decision-making [9,11,12,13,21,62,63,64]. Animal studies have shown that repeated restraint stress in both mice and rats decreases AEA content but increases 2-AG content in the medial PFC [47,51]. However, neither single nor repeated swim stress exposure altered PFC 2-AG content in mice [65]. In addition, maternal separation in rats did not alter PFC AEA and 2-AG contents compared to controls at any tested age (Postnatal day 2–70) [66]. Therefore, stress exposure in rodents could affect AEA and 2-AG contents in the medial PFC only. Twenty-four hours of alcohol exposure via a liquid diet in rats caused a decrease in 2-AG content but no change in AEA content in the PFC [55]. In addition, AEA content in the cortex increased after mice were exposed to CIE for 72 h [52]. Although our results showed that neither mSPS, CIE, nor co-occurring conditions affected AEA or 2-AG contents, or NAPE-PLD or α-DAGL or FAAH or MAG-L or CB1R levels compared to controls, statistical analysis showed that AEA content was influenced by CIE exposure independently of mSPS condition, which indicates that AEA content in the PFC, one of the brain regions in the reward pathway, is disrupted largely by CIE exposure in mice. In addition, AEA and 2-AG levels are largely influenced by circadian rhythms, such that AEA levels in the PFC in rodents are lower during their light cycle (inactive phase) than their dark cycle (active phase) [67,68]. Therefore, AEA levels could have been too low to detect significant differences among groups in this study as the animals were euthanized during their light cycles.

The STR is another main reward-associated brain region that plays an important role in regulating motivation, habitual action and learning, goal-directed behaviors, and cognition, and studies have shown that alcohol exposure alters striatal function [11,12,20,64,69,70,71,72,73,74]. Our results showed that both AEA and 2-AG content increased in the anterior STR after mice were exposed to both mSPS and CIE compared to mice exposed to mSPS only. In contrast, both NAPE-PLD, α-DAGL, FAAH, MAG-L, and CB1R levels remained unchanged among groups after mice were exposed to mSPS and/or CIE. Statistical analysis showed an effect of CIE on AEA and 2-AG levels, which suggest that striatal AEA and 2-AG content increases observed in mSPS-CIE mice are driven by CIE exposure. Besides the general roles of AEA on the central nervous system, a study in mice has shown that AEA also regulates 2-AG content, metabolism, and physiological effects in the STR [75]. This suggests that AEA content may negatively correlate with 2-AG content in the STR. However, our results showed that both AEA and 2-AG levels were increased in the anterior STR after mice were exposed to both mSPS and CIE. This finding indicates that a combination of traumatic stress and chronic alcohol exposure disrupts the regulation of 2-AG content. In addition, even though both the levels of NAPE-PLD, α-DAGL, FAAH and MAG-L remained unchanged, their activities could be increased (NAPE-PLD and α-DAGL) and decreased (FAAH and MAG-L) after exposures to both mSPS and CIE in mice [47,52]. Research has shown that 2 cycles of CIE causes an increase in 2-AG content while maintaining AEA content levels [53]. Our results showed 2-AG and AEA content did not change in Control-CIE mice, which could be due to exposure to additional cycles of CIE exposure. We previously reported that the combined exposure of mSPS and acute alcohol administration decreased CB1R levels but was unchanged in either mSPS or acute alcohol administration condition alone in the mouse anterior STR compared to control groups [20]. Our results in this study agreed our previous work that CB1R was unchanged in mSPS-Air mice. However, in the present study, CB1R levels did not change after mSPS and CIE exposure, which could be due to different alcohol regimens (acute (11 d) vs. chronic (4 wks including withdrawal)) and/or routes of administration (2.0–2.5 g/kg of 20% ethanol; intraperitoneal vs. 95% ethanol vapor).

The NAc is involved in both the reward pathway and limbic system activity with primary roles not only in reward seeking and reinforcement (both positive and negative) [17,76,77,78], but also stress and anxiety [79,80,81,82,83,84]. Little research has been done to investigate the effects of PTSD or PTSD-like stress on the NAc. One clinical research study found that PTSD patients had reduced activation in the NAc compared to healthy individuals on reward responsivity during a decision-making task [80]. In animal research, rodent studies showed reduced cFos expression [83], reduced dopamine release [85], and reduced dopamine, including its metabolites content and dopamine 2 receptor density [73], as well as enhanced dopamine transporter density [73] in the NAc after SPS compared to controls. In alcohol studies, previous research has shown unaltered AEA with increased 2-AG content in the NAc of rats self-administering alcohol [86,87], which is an alcohol administration method different from our method in this study. Our current study showed that AEA and 2-AG content, and their degradatory-hydrolytic enzymes levels did not change after mice were exposed to mSPS or CIE or both, which could be due to low to moderate CB content [17,88] in the NAc. The animals in this study were euthanized during their light cycles, and AEA levels in the NAc are lower during the light cycle than the dark cycle [67,68], which could account for no detectable differences in AEA levels among groups.

The HC, one of the primary structures in the limbic system, regulates learning and memory as well as HPA axis inhibition [10,12,14,59,64,89,90,91,92,93], and specifically, the dorsal HC modulates cognitive function [94]. Our results showed that mice exposed to CIE only had a higher AEA content compared to other groups (Control-Air, mSPS-Air, or mSPS-CIE) studied here, which suggests a CIE-driven effect in hippocampal AEA content. In addition, an increase in AEA content could be caused by increases in NAPE-PLD and decreases in FAAH activity even though our results showed the NAPE-PLD and FAAH levels were unchanged [46,52]. Our results parallel those in other published studies, which showed AEA content was increased, but 2-AG content was unchanged in the HC 4–24 h after postnatal day 7 mice were treated with ethanol [95]. In addition, AEA content increased after rats were exposed to CIE followed by 40 d of alcohol withdrawal [96]. Animal research has also shown that both acute and chronic stress decreased AEA content in the HC [24,44,51]. However, our results showed AEA content did not change in mSPS-Air mice, which could be due to differences in stress paradigms. Other rodent studies have shown that chronic restraint, as well as social defeat stress, caused an increase in 2-AG content in the HC [44], while chronic unpredictable stress caused a decrease in 2-AG content [97]. Therefore, both duration and types of stressors could affect 2-AG content. Statistical analysis showed a main effect of mSPS exposure on 2-AG content, which suggests that 2-AG levels were preferentially influenced by mSPS exposure independently of CIE condition. The 2-AG content was unchanged in mSPS-CIE mice compared to the remainder of the groups, which indicates that both traumatic stress and chronic alcohol exposure act together to modulate AEA content in the dorsal HC.

The AMYG, another primary structure of the limbic region, plays an important role in emotion and fear [11,59,61,98,99]. Our results in this study showed AEA content was increased in the AMYG in Control-CIE mice compared to Control-Air or mSPS-CIE mice. Research has shown that rats exposed to CIE for 3 weeks followed by different durations of alcohol withdrawal decreased 2-AG content while AEA content was unchanged in the central AMYG [100]. Another research study has also shown that AEA content in the AMYG was decreased while 2-AG content was unchanged after rats were exposed to CIE for 6 weeks [54]. Our results contradict that report, which could be due to different durations of CIE exposures with no extra alcohol withdrawal after 4 cycles of CIE. An increase in AEA content could also be caused by a decrease in FAAH activity or an increase in NAPE-PLD in the absence of altered protein levels [42,43,47,52]. In addition, the statistical analysis in this study showed both a main effect of mSPS exposure and an interaction of mSPS and CIE exposure on amygdalar AEA content, which suggests that mSPS and CIE act together to regulate AEA content. Even though amygdalar 2-AG content was unchanged, statistical analysis showed an mSPS effect on 2-AG content, which suggests a stress-driven effect that is independent of CIE exposure.

## 4. Materials and Methods

### 4.1. Animals

Adult male C57Bl/6 mice were 10–12 weeks old at the time of the study. Animals were bred in-house in a Wayne State University (WSU) climate-controlled vivarium under a 12-h light/dark cycle (lights on at 6 am). Mice were group housed (2–5 mice/cage) in standard microisolator polycarbonate cages with *ad libitum* access to food and water, except during the mSPS procedure. All procedures were conducted according to the National Institute of Health Office of Laboratory Animal Welfare Guide for the Care and Use of Laboratory Animals [101] approved by the WSU Institutional Animal Care and Use Committee. The sample size for this study was based on a power analysis using an estimate of variance obtained from our previous studies. This analysis yielded the estimated number of animals that must be used to have a power of >88% (α = 0.05) with significance set at *p* < 0.05.

### 4.2. Mouse Single-Prolonged Stress (mSPS)

**Scheme 1:**
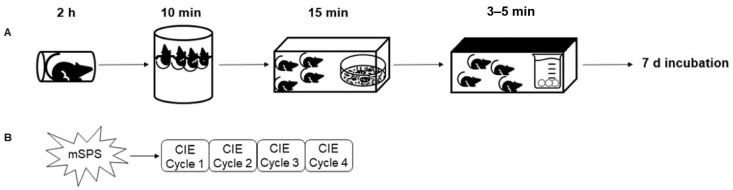
Schematic overview of (**A**) the mouse single prolonged stress (mSPS) paradigm, (**B**) mSPS-CIE consecutive cycles paradigm. After mSPS paradigm and 7 days incubation, mice were exposed to 4 consecutive cycles of CIE exposure.

Mice were exposed to mSPS as described previously [9,20,57] (See Scheme 1A).

Briefly, mice were exposed to consecutive stressors in a 2.5-h time frame in the following order: 2-h individual restraint in 50 mL conical tubes (holes made along the tubes for ventilation), then 10 min group (3–4 mice/group) forced swim in a 4-L beaker with water at 26–28 °C, followed by 15 min exposure to odor of soiled rat bedding (a predator scent), and finally diethyl ether exposure until unconscious. Mice were transferred to a clean microisolator polycarbonate cage after regaining consciousness. During mSPS exposure, groups of control mice receiving no mSPS exposure were weighed and handled in another room. After the mSPS exposure session, both control and mSPS-exposed mice were group housed, according to their original social groupings, in clean cages (4–5 mice/cage) and left undisturbed for 7 d with access to food and water and daily health checks.

### 4.3. Chronic Intermittent Ethanol (CIE) Vapor Exposure

After mSPS exposure including the 7-d incubation period, mice were randomly assigned to 1 of 4 groups (Control-Air, Control-CIE, mSPS-Air, or mSPS-CIE) and exposed to CIE or air on the 8th day, which was adapted from published methods [102] (See Scheme 1B). In this paradigm, 95% ethanol mixed with air or air alone was continuously pumped into plexiglass inhalation chambers (Plas Lab, Lansing, MI, USA) at a rate of 10 L/min in which homecages were placed. Mice were exposed to air or ethanol vapor for 16 h followed by 8 h room air exposure (an ethanol abstinence period) for 4 consecutive days. This was considered to be one cycle, and mice were exposed to ethanol vapor or air for 4 consecutive cycles. In order to stabilize blood ethanol concentrations and initiate ethanol intoxication, mice were co-administered 1 mmol/kg of pyrazole, an alcohol dehydrogenase (Chem-Impex International, Wood Dale, IL, USA), and 1.6 g/kg of 20% *w/v* ethanol (CIE exposure mice) or 10 mL/kg of saline (air exposure mice) intraperitoneally before being placed into the inhalation chambers. Plasma samples were collected every week to measure blood ethanol concentrations (BEC), and BEC levels above 175 mg/dL were considered intoxicated. Average BECs across the 4 cycles for the Control-CIE and mSPS-CIE mice were 195.6 mg/dL and 192.2 mg/dL, respectively. Average BEC levels for Control-Air and mSPS-Air mice were undetectable.

### 4.4. Brain Tissue Dissection

Mice were euthanized by cervical dislocation without anesthetics 24 h after the last day of CIE exposure. Mouse brains were sliced into 2-mm-thick coronal sections using an ice-cold mouse brain slicer matrix (Zivic Instrument, Pittsburgh, PA, USA). Bilateral brain regions were micro-dissected from the coronal sections using a tissue biopsy punch with plunger (Miltex, York, PA, USA) (1.5-mm diameter for PFC, anterior STR, and dorsal HC; 1.0-mm for NAc and AMYG) and stored at −80 °C until ultra-high-performance liquid chromatography tandem mass spectrometry (UHPLC-MS/MS) or immunoblotting analyses.

### 4.5. AEA and 2-AG Extraction for UHPLC-MS/MS Analysis

The sample extraction of AEA and 2-AG was adapted from published methods [103]. Twenty-seven of mouse brain tissue samples were weighed prior to extraction. One milliliter of 1:1 cold methanol (MeOH) (liquid chromatography-mass spectrometry (LC-MS) grade; Sigma-Aldrich, St. Louis, MO, USA) to acetonitrile (high performance liquid chromatography grade; Sigma-Aldrich) were added to each sample, and the sample was spiked with 2.5 fmol/μL of d_8_-AEA (Cayman Chemical, Ann Arbor, MI, USA) and 0.25 ρmol/μL of d_8_-2AG (Cayman Chemical) to account for any sample loss during the extraction process. Samples were then homogenized using a Branson Ultrasonics Sonifier S-250A homogenizer (Thermo Fisher Scientific, Waltham, MA, USA) for 20 s. After homogenization, sample homogenates were centrifuged for 20 min at 14,000× *g* at 4 °C. Supernatants were then transferred to a new microcentrifuge tube and evaporated under N_2_ gas at room temperature. Then, 200 μL of 30:70 LC-MS grade MeOH to H_2_O was added to resuspend each sample before loading them to a SPE cartridge. After samples were loaded on the SPE cartridge, 2 × 250 μL of LC-MS grade H_2_O were added to remove salt and other polar materials. After the washing steps, the analytes were eluted with 3 × 200 μL of 100% LC-MS grade MeOH and dried under N_2_ gas at room temperature before storage at −80 °C until use.

### 4.6. UHPLC-MS/MS

AEA and 2-AG analyses were performed on an UHPLC coupled to tandem positive electrospray ionization triple quadrupole MS (Shimadzu 8040) at the Lumigen Instrument Center, Department of Chemistry at WSU. An autosampler of the UPLC was set at 4 °C. Analysis setup was adapted from literature [104]. Mobile phases (A: 2 mM ammonium acetate in H_2_O; B: 2 mM ammonium acetate in MeOH) were used with the flow rate set at 0.5 mL/min. The gradient was set as follows to elute analytes of interest: 0.0–0.5 min 75% B, 0.5–5.0 min to 79%, 5.0–5.5 min to 90% B, and 5.5–6.5 min at 75% B. Analytes in the samples were separated using an ACQUITY BEH C18 liquid chromatography column with inner diameter of 100 mm × 2.1 mm and 1.7 μm particle size (Waters, Milford, MA, USA) and thermostat-controlled at 60 °C.

During the analyses, a standard curve of AEA (0–100 fmol/μL) and 2-AG (0–25 ρmol/μL) containing the internal standards of 0.25 ρmol/μL d_5-_2AG, 0.25 ρmol/μL d_8_-2AG, 2.5 fmol/μL d_4_-AEA, and 2.5 fmol/μL d_8_-AEA in 1:3 LC-MS grade H_2_O to MeOH (Cayman Chemical) was run before and after every 15 samples. The internal standards of d_5_-2AG and d_4_-AEA were used to correct for instrumental variation, whereas, d_8_-2AG and d_8_-AEA were used to assess sample degradation or loss from the extraction process. Samples were reconstituted with 0.25 ρmol/μL of d_5_-2AG and 2.5 fmol/μL of d_4_-AEA in 1:3 LC-MS grade H_2_O to MeOH.

### 4.7. Protein Extraction for Immunoblot Analysis

A unilateral brain tissue punch (24 mouse brain tissue) was homogenized using a motorized tissue grinder (Fisher Scientific, Waltham, MA, USA) in lysis buffer (20 mM Tris pH 7.5 (Fisher Scientific), 150 mM sodium chloride (Fisher Scientific), 2 mM EDTA pH 8.0 (Sigma-Aldrich), 1% triton X-100 (Sigma-Aldrich), 10% glycerol (Sigma-Aldrich), 1% protease inhibitors (Sigma-Aldrich), 2% phosphatase inhibitor cocktail II (Sigma-Aldrich) and 2% phosphatase inhibitor cocktail III (Sigma-Aldrich)) prepared in dH_2_O. Samples were then centrifuged at 10,000× *g* for 10 min at 4 °C. A Pierce 660 nm protein assay with bovine serum albumin standard (Thermo Fisher Scientific) was used to measure protein concentrations in the supernatant.

### 4.8. Immunoblotting

Proteins of interest in the PFC, anterior STR, NAc, dorsal HC, and AMYG were analyzed using immunoblotting. Protein extracts (PFC and anterior STR: 10 μg; NAc and dorsal HC: 20 μg; AMYG: 25 μg) from each sample were loaded and separated on a 4–12% Bis-Tris gel with 20X MOPS SDS running buffer (Thermo Fisher Scientific) and transferred to a nitrocellulose membrane using 20× transfer buffer (Thermo Fisher Scientific) on a semi-dry transfer apparatus (Thermo Fisher Scientific) at 15V for 40 min at room temperature. Membranes were then washed 3 × 10 min with 1X tris buffered saline with 0.1% tween 20 (TBST) and blocked with 5% non-fat dry milk in 1X TBST (anti-FAAH or MAG-L) or 5% bovine serum albumin (BSA) in 1X TBST (anti-α-DAGL and anti-CB1R) or 5% non-fat dry milk with 1% BSA in 1X TBST (anti-NAPE-PLD) for 1 h. Membranes were then washed with 1X TBST. Primary antibodies were added separately to the membranes followed by incubation overnight at 4 °C. Membranes were exposed to actin, the loading control, for 1 h at room temperature. After incubation with primary antibodies, membranes were washed 3 × 10 min with 1X TBST and incubated with corresponding secondary antibodies (anti-mouse for anti-FAAH or anti-actin, and anti-rabbit for anti-MAG-L, anti-α-DAGL, anti-NAPE-PLD, and anti-CB1R) for 1 h (FAAH, MAG-L, α-DAGL, NAPE-PLD, or anti-CB1R) or 30 min (actin) at room temperature. The following primary and secondary antibodies were applied individually, and the membrane was stripped with Restore^TM^ Plus Western Blot Stripping Buffer (Thermo Fisher Scientific) according to the manufacture protocol between antibodies. Antibody concentrations were: 1:2500 (PFC, anterior STR, AMYG) and 1:5000 (NAc and dorsal HC) anti-NAPE-PLD (Novus Biologicals, Englewood, CO; catalog number: NB110-80070), 1:1000 anti α-DAGL (Cell Signaling Technology, Danvers, MA, USA; catalog number 13626S), 1:2000 anti-FAAH (Cell Signaling Technology; catalog number: 2942S), 1:300 anti-MAG-L (Cayman Chemical; catalog number: 100035), 1:500 anti-CB1R (Cell Signaling; catalog number: 93815S), 1:5000 anti-actin (Sigma-Aldrich; catalog number: A5060), 1:20,000 goat anti-mouse (Cell Signaling; catalog number: 7076) for anti-FAAH, 1:10,000 goat anti-rabbit (PFC, anterior STR, AMYG) and 1:30,000 (NAc and dorsal HC) (Cell Signaling; catalog number: 7074) for anti-NAPE-PLD, 1:10,000 goat anti-rabbit anti-α-DAGL(Cell Signaling), 1:600 goat anti-rabbit for anti-MAG-L, 1:1000 for anti-CB1R and 1:2000 goat anti-mouse for anti-actin. Proteins of interest on the immunoblots were detected using enhanced chemiluminescence, and molecular weights were determined using molecular weight standards (Thermo Fisher Scientific).

### 4.9. Data Analysis and Statistics

The analyses of UPLC-MS/MS of AEA and 2-AG and immunoblotting of FAAH, MAG-L, NAPE-PLD, α-DAGL, and CB1R were calculated using MS Excel, and the graphing and statistical analysis were conducted using GraphPad 8.3 (San Diego, CA, USA) and Statistica 6.0 (Tulsa, OK, USA). For the UPLC-MS/MS analysis, standard curves and the analyte in the samples were normalized to the corresponding internal standard values (d_5_-2AG for 2-AG and d_4_-AEA for AEA). AEA and 2-AG contents were then calculated by using the standard curve, normalized to the weight of mouse brain tissues, and corrected for sample degradation/loss from the extraction process using the values of d_8_-AEA for AEA and d_8_-2AG for 2AG. For immunoblotting analysis, FAAH, MAG-L, and actin protein levels were quantified using Image J, and the proteins of interest were normalized to actin. The two-sided Grubbs’ test was used to detect any outliers using α = 0.05 as the criterion for significance. Two-way ANOVA was conducted on each measure with mSPS and CIE as main factors, followed by Tukey HSD post hoc analysis, as appropriate. Results were reported as mean ± SEM and compared using α = 0.05 and *p* ˂ 0.05 as the criterion for statistical significance.

## 5. Conclusions

In conclusion, this current study aimed to evaluate the effect of traumatic stress and chronic ethanol exposure on the CB system, including AEA and 2-AG content and FAAH, MAG-L, NAPE-PLD, α-DAGL, and CB1R levels, in limbic and reward brain regions. We show that AEA content was affected by CIE exposure independently of mSPS exposure in the PFC. In addition, mSPS-CIE comorbidity altered AEA and 2-AG content in the anterior STR. However, neither mSPS nor CIE affected the CB system in the NAc, which could be due to low to moderate amounts of AEA or 2-AG in that region. It could also be due to the time of day when animals were euthanized. In the central brain regions of the reward pathway, our novel findings indicate that alcohol affects AEA content in the PFC, and traumatic stress and chronic alcohol exposure/withdrawal comorbidity affects AEA or 2-AG levels in the anterior STR, which appears to be driven by alcohol. In the limbic system, other novel findings show mSPS and CIE interaction and an mSPS main effect on AEA content with increased dorsal HC AEA levels in Control-CIE mice compared to Control-Air, mSPS-Air, or mSPS-CIE mice, as well as an mSPS main effect on 2-AG content. Our findings also show mSPS and CIE interaction and an mSPS main effect on AEA content with increased AEA levels in the AMYG in Control-CIE mice compared to Control-Air or mSPS-CIE mice. In addition, there is an mSPS main effect on 2-AG content in the AMYG. Our findings suggest that changes in AEA content in the dorsal HC and AMYG, the two primary structures of the limbic system, are driven independently by mSPS exposure or mSPS-CIE co-exposure, while 2-AG content in these brain regions is mainly affected by mSPS. Studies have shown that chronic stress exposure, CIE exposure or mSPS/acute alcohol exposure affect CB1 expression/density [20,24,52,53]. However, we did not see any changes in CB1R protein levels among groups after mSPS and CIE, which could be due to different stressor types or routes of alcohol administration or durations of alcohol exposure. Results from this current study provide support to explore additional brain regions such as the hypothalamus, which is a brain region that mediates stress-related and alcohol-related studies [44,105]. It would also be informative to quantify any changes of AEA and 2-AG levels in blood and central spinal fluid after mSPS and/or CIE as it has been shown that plasma AEA and 2-AG levels vary after stress in clinical studies [40,106]. Together, these data will guide the development of neurotherapeutic approaches to mitigate changes in AEA and 2-AG levels caused by both traumatic stress and chronic ethanol exposure, which we demonstrate uniquely impact reward and limbic regions.

## Data Availability

Data are available upon request.

## Supplementary Materials

The following are available online, Figure S1: N-acyl phosphatidylethanolamine phospholipase (NAPE-PLD) levels in different brain regions of interest after mSPS/Control or Air/CIE exposures; Figure S2: α-Diacylglycerol lipase (α-DAGL) levels in different brain regions of interest after mSPS/Control or Air/CIE exposures; Figure S3: Cannabinoid 1 receptors (CB1R) levels in different brain regions of interest after mSPS/Control or Air/CIE exposures.

## Sample Availability

Samples of the compounds are not available from the authors.
