# Supplementary material for: Traumatic Stress, Chronic Ethanol Exposure, or the Combination, Alter Cannabinoid System Components in Reward and Limbic Regions of the Mouse Brain
Source: Molecules. 2021 Apr 6;26(7):2086. doi: 10.3390/molecules26072086 (PMC8038692; doi:10.3390/molecules26072086)
Supplement: Supplementary file 1 [file molecules-26-02086-s001.pdf]

Supplementary Materials

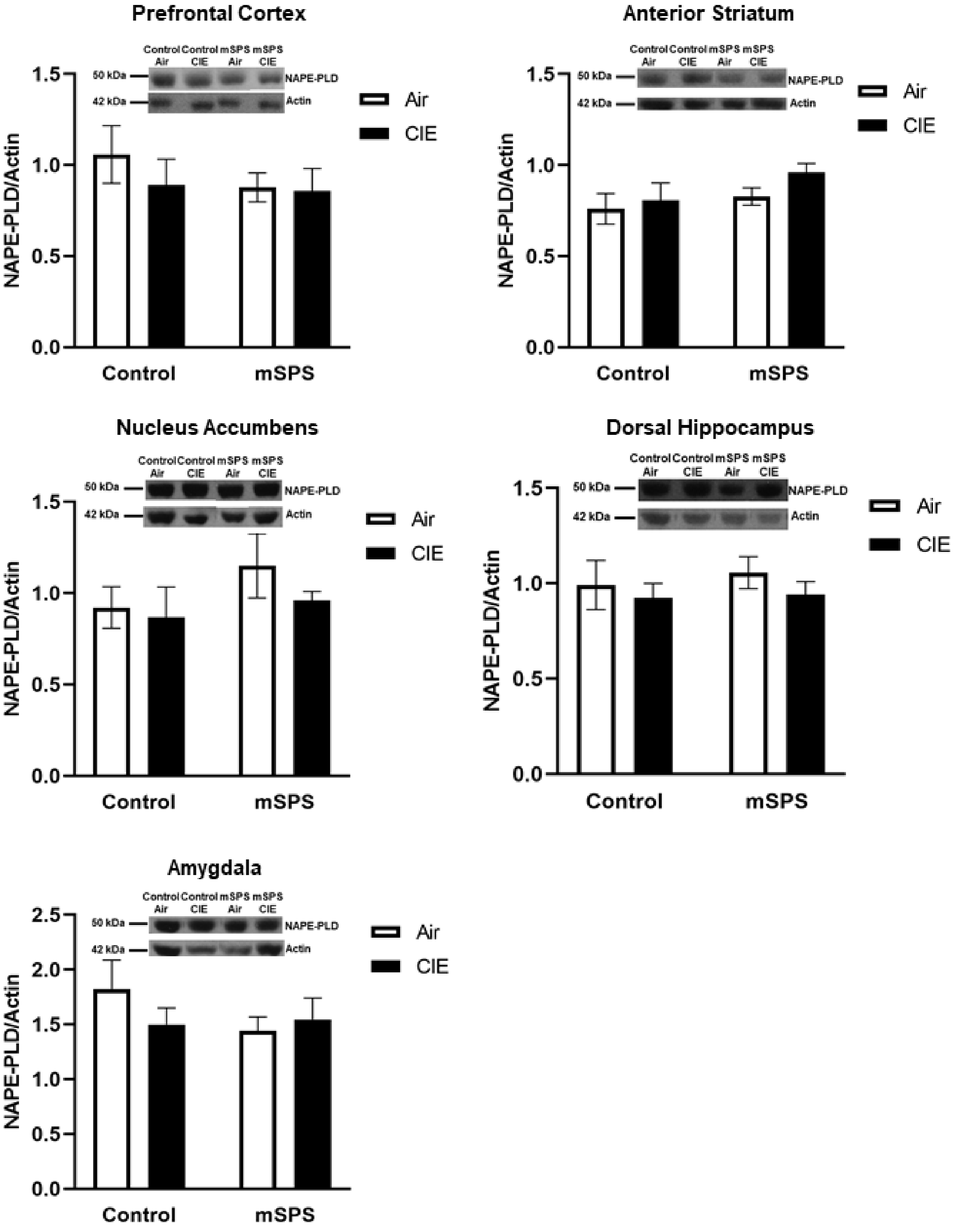

Figure S1. N-acyl phosphatidylethanolamine phospholipase (NAPE-PLD) levels in different brain regions of interest after mSPS/Control or Air/CIE exposures. Average NAPE-PLD levels in the A) prefrontal cortex (Control-Air: n = 7; Control CIE: n = 4; mSPS-Air: n = 5; mSPS-CIE: n = 8), B) anterior striatum (Control-Air:

n = 7; Control CIE: n = 4; mSPS-Air: n = 5; mSPS-CIE: n = 8), C) nucleus accumbens (Control-Air: n = 7; Control CIE: n = 4; mSPS-Air: n = 5; mSPS-CIE: n = 8), D) dorsal hippocampus (Control-Air: n = 7; Control CIE: n = 4; mSPS-Air: n = 5; mSPS-CIE: n = 8), and E) amygdala (one sample was excluded after outlier analysis; Control-Air: n = 7; Control CIE: n = 4; mSPS-Air: n = 5; mSPS-CIE: n = 7) (Inset: representative immunoblotting sample images) did not change among groups after mSPS/Control or Air/CIE exposures. Data are mean ± SEM.

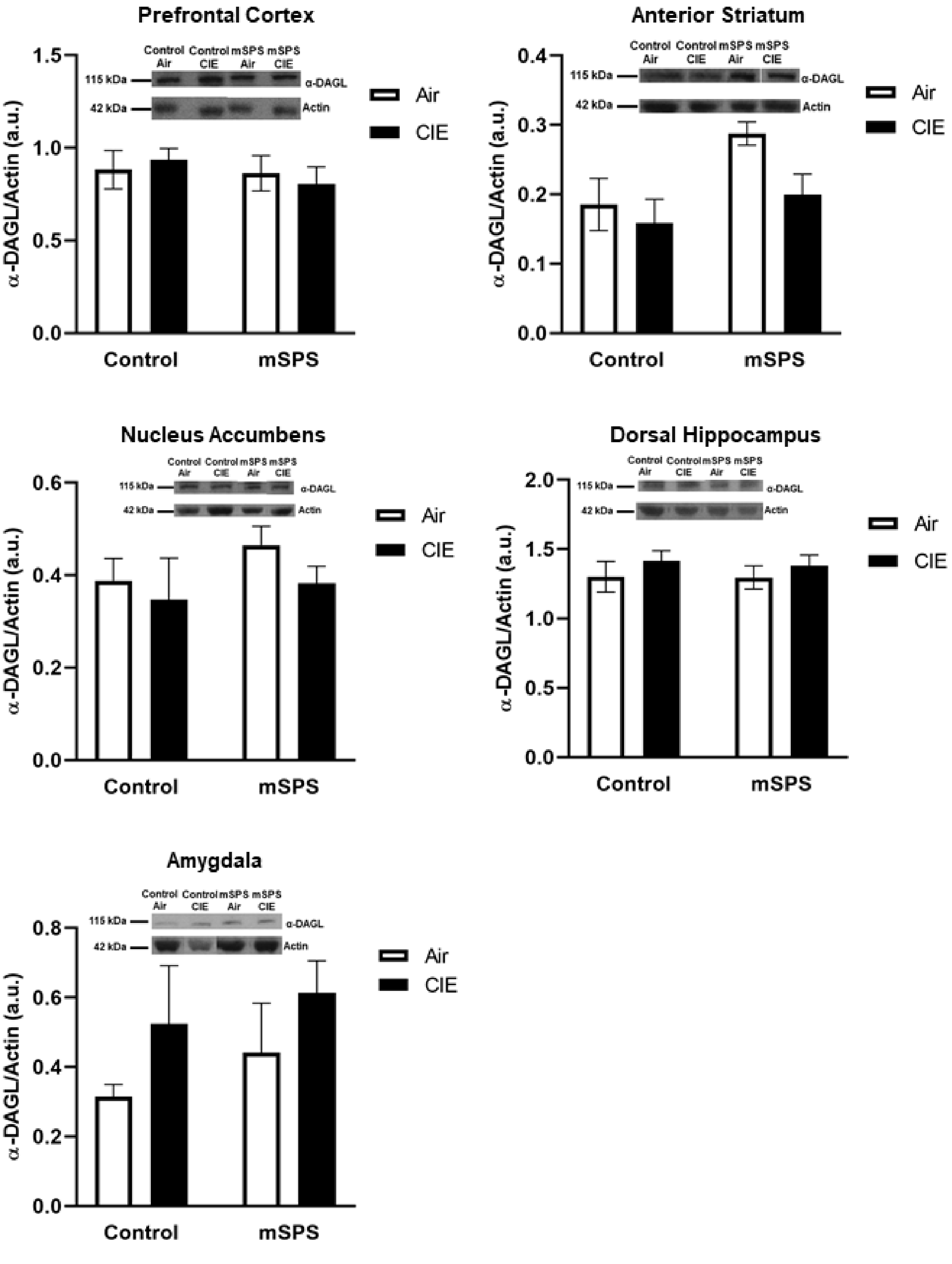

Figure S2. α-Diacylglycerol lipase (α-DAGL) levels in different brain regions of interest after mSPS/Control or Air/CIE exposures. Average α-DAGL levels in the A) prefrontal cortex (Control-Air: n = 7; Control CIE: n = 4; mSPS-Air: n = 5; mSPS-CIE: n = 8), B) anterior striatum (Control-Air: n = 7; Control CIE: n = 4; mSPS-

Air: n = 5; mSPS-CIE: n = 8), C) nucleus accumbens (Control-Air: n = 7; Control CIE: n = 4; mSPS-Air: n = 5; mSPS-CIE: n = 8), D) dorsal hippocampus (one sample was excluded after outlier analysis; Control-Air: n = 7; Control CIE: n = 4; mSPS-Air: n = 5; mSPS-CIE: n = 7), and E) amygdala (one sample was excluded after outlier analysis; Control-Air: n = 6; Control CIE: n = 4; mSPS-Air: n = 5; mSPS-CIE: n = 8) (Inset: representative immunoblotting sample images) did not change among groups after mSPS/Control or Air/CIE exposures. Data are mean ± SEM.

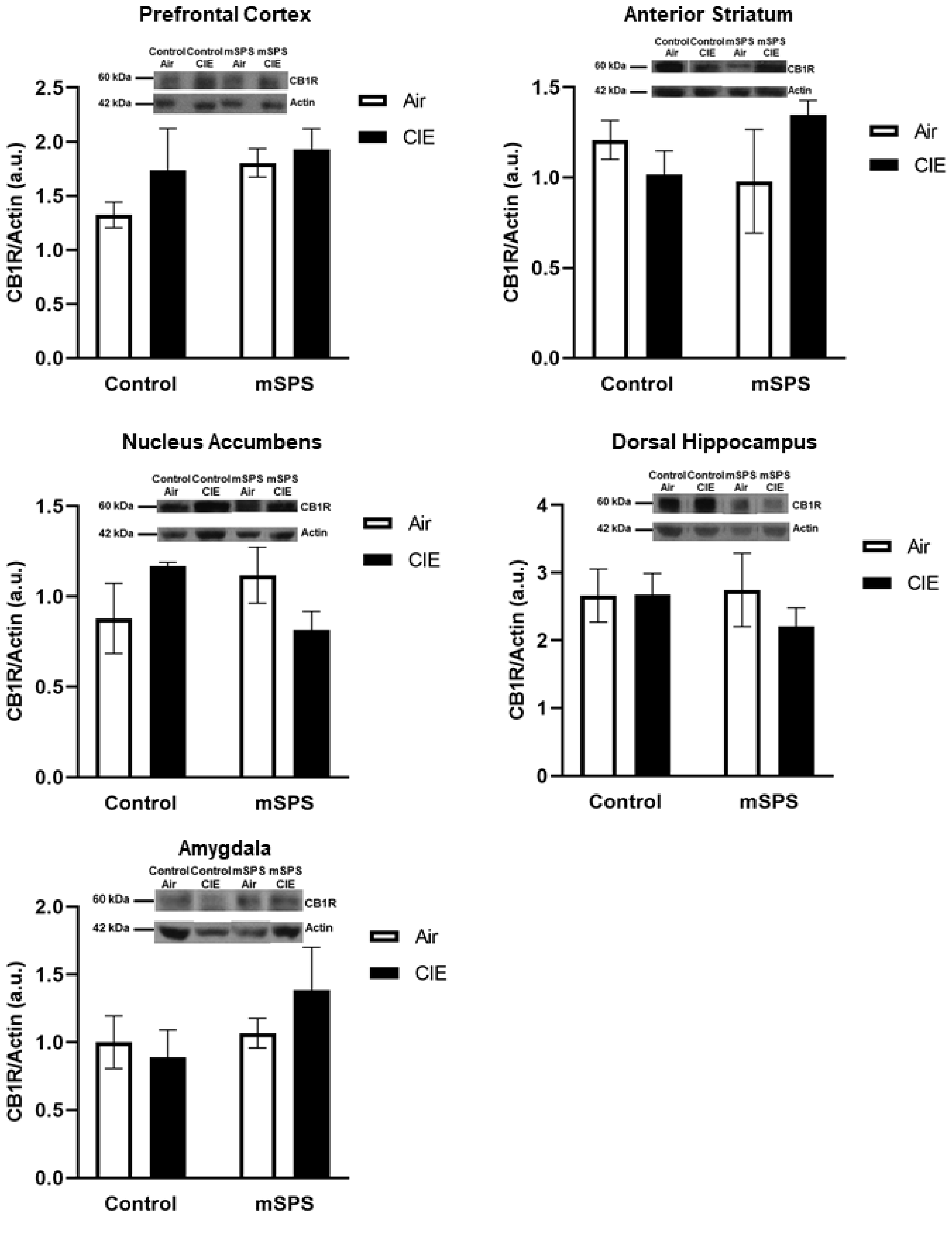

Figure S3. Cannabinoid 1 receptors (CB1R) levels in different brain regions of interest after mSPS/Control or Air/CIE exposures. Average CB1R levels in the A) prefrontal cortex (Control-Air: n = 7; Control CIE: n = 4; mSPS-Air: n = 5; mSPS-CIE: n = 8), B) anterior striatum (one sample was excluded after outlier analysis; Control-Air: n = 7; Control CIE: n = 4; mSPS-Air: n = 5; mSPS-CIE: n = 7), C) nucleus accumbens (one sample

was excluded after outlier analysis; Control-Air: n = 7; Control CIE: n = 3; mSPS-Air: n = 5; mSPS-CIE: n = 8), D) dorsal hippocampus (Control-Air: n = 7; Control CIE: n = 4; mSPS-Air: n = 5; mSPS-CIE: n = 8), and E) amygdala (Control-Air: n = 7; Control CIE: n = 4; mSPS-Air: n = 5; mSPS-CIE: n = 8) (Inset: representative immunoblotting sample images) did not change among groups after mSPS/Control or Air/CIE exposures. Data are mean ± SEM.
